# Identification of metabolic signatures of immune response following mRNA and inactivated vaccines against COVID-19: a systematic review

**DOI:** 10.3389/fimmu.2026.1783878

**Published:** 2026-02-25

**Authors:** Andrzej Wasilewski, Agata Serrafi

**Affiliations:** 1 Student Scientific Association of Medical Chemistry and Immunochemistry, Wroclaw Medical University, Wrocław, Poland; 2 Department of Immunochemistry and Chemistry, Wroclaw Medical University, Wrocław, Poland

**Keywords:** biomarkers, COVID-19 vaccine, immune response, kynurenine, metabolomics

## Abstract

**Background:**

Metabolomic profiling offers insights into immune responses, yet a synthesis of systemic metabolic changes after COVID-19 vaccination is lacking. This review aims to characterize vaccination-induced metabolomic alterations and identify correlative biomarkers of responsiveness.

**Methods:**

Following PRISMA 2020 guidelines (PROSPERO 1181037), four databases (PubMed, Embase, Scopus, Web of Science) were searched for studies using LC-MS, GC-MS, or NMR to analyse venous blood after COVID-19 vaccination. Inclusion criteria focused on original human studies. Risk of bias was assessed using ROBINS-I and RoB 2.

**Results:**

Ten studies (n > 1,200) evaluating mRNA and inactivated vaccines were included. Vaccination consistently altered amino acid pathways, specifically glutamine, phenylalanine, and tryptophan. Early activation of the kynurenine pathway (1–2 days post-dose) emerged as a predictor of stronger antibody responses. Inactivated vaccines triggered a “Warburg-like” metabolic switch, characterized by increased glycolysis and reduced TCA intermediates. Lipidomic changes were prominent; high baseline ceramides predicted low response, while sphingomyelins and short-chain fatty acids associated with positive immunity. Most studies showed a moderate risk of bias due to *post-hoc* grouping and confounding factors.

**Conclusions:**

COVID-19 vaccination induces reproducible changes in amino acid, energy, and lipid metabolism. Kynurenine activity, baseline amino acids, and sphingolipid signatures are potential predictors of vaccine efficacy, supporting personalized immunization strategies.

## 1 Introduction

The SARS-CoV-2 pandemic, which has been ongoing since late 2019, has been the greatest health challenge of the 21st century, causing over 700 million cases and approximately 7 million deaths worldwide (1). COVID-19 vaccinations have played a key role in controlling the epidemic, significantly reducing hospitalizations, deaths, and virus transmission (2). By August 2024, more than 13.53 billion doses of vaccine had been administered worldwide, resulting in at least one dose being administered to 70.6% of the global population (3). Widely used preparations belong to different technological platforms: mRNA vaccines (e.g., BNT162b2 from Pfizer-BioNTech and mRNA-1273 from Moderna), vector vaccines (e.g., ChAdOx1 nCoV-19 from AstraZeneca and Ad26. COV2.S from Janssen), inactivated (e.g., CoronaVac from Sinovac), and subunit protein (e.g., NVX-CoV2373 from Novavax) (4).

Despite the high efficacy of vaccines, significant heterogeneity in immune response has been observed among different individuals, with 10–20% of vaccine recipients developing poor neutralizing antibody production or cellular immune response (5). Factors such as age, comorbidities, obesity, and immunosuppression affect the effectiveness of vaccination (6, 7). In addition, vaccinations induce transient inflammation, manifested by post-vaccination symptoms, which may vary depending on the type of preparation (8).

Vaccinations trigger multilevel immune responses ranging from immediate innate immune signals to long-lasting humoral and cellular responses, and their mechanisms can be studied using a “systems vaccinology” approach, which uses omics techniques to identify early correlative biomarkers and regulatory mechanisms of the post-vaccination response (9, 10). This systems approach has revealed that host metabolism is closely linked to the nature and strength of the immune response after vaccination.

Previous studies on the metabolomic response to COVID-19 vaccination suggest significant but heterogeneous metabolic changes depending on the vaccine platform and the time point of measurement (11). For example, studies involving inactivated vaccines (CoronaVac) have shown changes in TCA cycle metabolism, amino acids, and microbiota-related metabolism, with correlations to antibody levels (12), while studies on mRNA vaccines (BNT162b2) have indicated differences in amino acid and lipid profiles between “high” and “low” responders, with ceramides and certain amino acids proposed as potential markers (11, 13).

Despite a growing number of individual studies demonstrating specific metabolomic changes following COVID-19 vaccination, there is a lack of systematic synthesis of these data. Existing studies differ in vaccine platforms, populations, analytical methods, and time points, making it impossible to identify common patterns and potential biomarkers. This systematic review, conducted in accordance with the PRISMA 2020 guidelines (14), aims to comprehensively evaluate data from peripheral blood metabolomic analysis following COVID-19 vaccination in order to: characterize vaccination-induced metabolomic changes; link them to immune response and inflammatory regulation; identify potential correlative biomarkers.

## 2 Materials and methods

### 2.1 Registration and protocol

This systematic review was conducted in accordance with a previously developed protocol. Both the protocol and the systematic review were registered in PROSPERO (No. 1181037). The review was conducted in accordance with the PRISMA 2020 guidelines (14).

### 2.2 Eligibility criteria

The inclusion and exclusion criteria for studies were determined using the PICOS framework:

*Population (P).*

*Inclusion Criteria*: Studies involving human participants who received any type of COVID-19 vaccine. Participants must not have an active SARS-CoV-2 infection at the time of sampling. Only studies analysing venous blood samples (serum, plasma, or whole blood) collected after vaccination will be included.

*Exclusion Criteria:* Studies using non-human models (animal, cell lines, *in vitro*, in silico), studies of infection without vaccination, studies based on biological fluids other than blood. Studies involving participants with chronic diseases will be excluded unless a healthy, vaccinated control group is analysed separately.

*Intervention (I).*

Intervention is the administration of any COVID-19 vaccine. This includes, but is not limited to, vaccines: mRNA-based (e.g., BNT162b2, mRNA-1273); vector-based (e.g., ChAdOx1 nCoV-19, Ad26.COV2.S); inactivated (e.g., CoronaVac, BBIBP-CorV); subunit protein (e.g., NVX-CoV2373).

*Comparison (C).*

Comparisons may include: pre-vaccination samples from the same individuals, unvaccinated control groups, different types of vaccines, and different time points after vaccination.

*Outcomes (O).*

*Primary Results:* Changes in metabolomic profiles (metabolites, lipidome, metabolic pathways) in venous blood after vaccination.

*Secondary Outcomes:* Data evaluating the immune response to vaccination. Identification of specific metabolites or pathways associated with immune response or adverse effects. Differences between vaccine types or sampling time points.

*Analytical Techniques:* Interest is in studies using LC-MS, GC-MS, CE-MS, NMR, and other analytical methods to characterize the metabolome.

*Study Type (S).*

*Inclusion Criteria:* Original studies, including clinical trials, cohort studies, cross-sectional studies, and experimental studies that report metabolomic data.

*Exclusion Criteria:* Reviews, meta-analyses, protocols, letters, conference abstracts, and preprints. Only peer-reviewed articles in English were included.

### 2.3 Search strategy

The following databases were searched: PubMed/MEDLINE, Embase, Scopus, and Web of Science. The studies were retrieved using a Boolean sequence: (metabolome OR metabolomics OR “metabolic profile” OR “metabolic profiling” OR lipidome OR lipidomics) AND (“COVID-19” OR “SARS-CoV-2”) AND (vaccine OR vaccination OR vaccinated OR immunization).

### 2.4 Study selection

The selection of studies was carried out by two independent reviewers. In order to optimize the abstract screening process in this review, automation based on a large language model (LLM) was implemented. This model, operationally integrated with the data management platform (Catchii) (15), acts as an inference engine. This model translates the protocol criteria (PICOS) into formal classification instructions, allowing for prioritized selection of material. Each record obtained using LLM was verified by a researcher and served only as support in the screening process.

*Stage 1:* Both reviewers will independently conduct a preliminary review of titles and abstracts for compliance with inclusion criteria.

*Stage 2:* Studies deemed potentially eligible will proceed to full-text assessment.

Disagreements in decisions were resolved by reaching a consensus.

### 2.5 Data collection process

The following information was extracted from the full texts of the included articles and supplementary materials: *Study characteristics*: author, year, country, study design; Population: sample size, demographics, vaccine type; *Samples*: sample matrix (serum, plasma, whole blood), time point of collection. *Analytical method*; *Main metabolomic results*: identified metabolites, altered pathways, clinical data, and additional data that may influence the interpretation of results, such as limitations, additional research methods. The visualization was created using the robvis tool (16).

### 2.6 Risk of bias

The risk of bias will be independently assessed by two reviewers. The ROBINS-I V2 (Risk Of Bias In Non-randomized Studies - of Interventions, Version 2) (17) will be used to assess non-randomized studies, while the Cochrane RoB 2 (Risk of Bias tool for randomized trials, Version 2) will be used to assess randomized controlled trials (RCTs) (18).

### 2.7 Data synthesis

The results were summarized narratively. Studies will be grouped by vaccine type, time point, and analytical technique used to synthesize consistent metabolic signatures.

## 3 Results

### 3.1 Characteristics of the included studies

**Figure 1:**
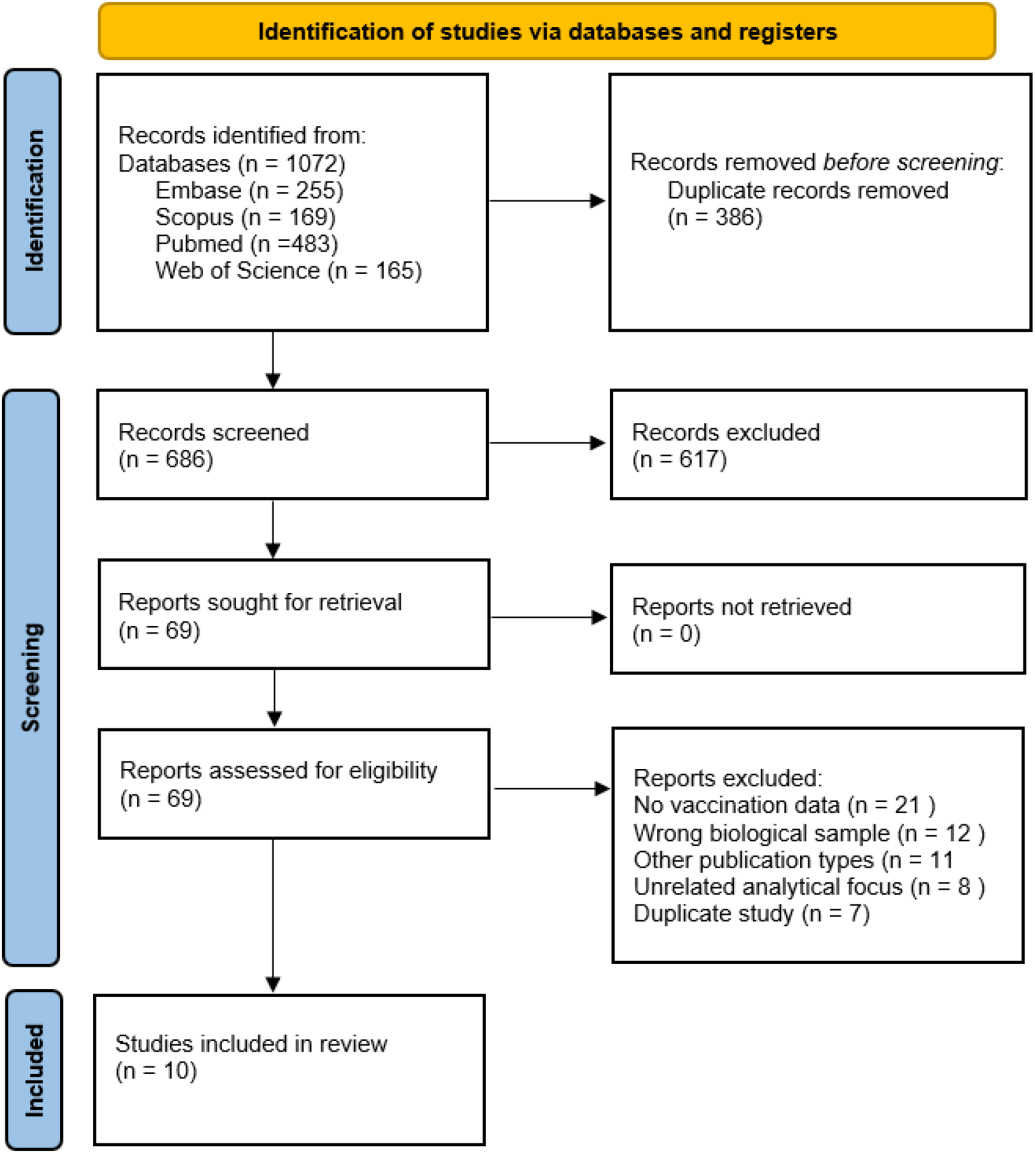
Prisma flow diagram.

Ten studies were included in the synthesis (11–13, 19–25) meeting the inclusion criteria, focusing on serum/plasma metabolomic profiling in response to COVID-19 vaccination. The study populations (total n > 1,200) mainly included healthy adults of various ages vaccinated with mRNA-based preparations (Pfizer/Comirnaty) and inactivated vaccines (Sinopharm, CoronaVac, BBIBP-CorV). Detailed results of individual stages of the research selection process are presented in **Figure 1**.

The studies were predominantly longitudinal in design, with sampling time points covering the period from baseline (pre-vaccination) to the short- and medium-term phases, reaching a maximum of 480 days after the first dose (21). The most commonly used analytical techniques were liquid chromatography-mass spectrometry (LC-MS) and nuclear magnetic resonance (NMR) spectroscopy.

The characteristics of the included studies are presented in **Table 1**. A summary of metabolomics data and key conclusions is presented in **Table 2**. An analysis of integrated key metabolomics data is summarised in **Table 3**.

**Table 1:** Study characteristics.

Ref.	Population size (n) and type	Vaccine (Type)	Key collection time points	Limitations/Comments
Abufares 2024 (19)	340 (Healthy): Pfizer (156), Sinopharm (107), Control (77). Age 8–90 years.	Pfizer (mRNA)/Sinopharm (Inactivated)	After a full cycle (2 doses).	No exact number of days after vaccination. No data on antibody titre.
Dagla 2022 (11)	58 (Healthy): Division into High vs Low Responders (NAbs on Day 22).	BNT162b2 (Pfizer) (mRNA)	Day 1 (before the first dose), Day 22 (before the second dose), 3 months after the first dose.	Classification based on neutralising antibody levels.
Ghini 2022 (20)	20 (Pilot): Naive (10) vs Recovered (10). Average age of Naive 41, Recovered 54.	Pfizer (mRNA)	6 fixed time points (short and medium term).	Small sample size (n=20). No data on inflammation markers.
He 2022 (13)	30 (Young adults aged 18–20). No history of SARS-CoV-2 infection.	CoronaVac (Inactivated)	Before vaccination (VB), 2 weeks after the second dose (SV).	Small research group. Restricted age range (18–20 years old).
Lang 2025 (21)	121 (Healthy): 61K/60M. Average age 37 ± 11 years. No previous infection.	Comirnaty (Pfizer) (mRNA, N = 30). Others: Vaxzevria + boosters.	Longitudinal (480 days): 26 time points. Short-term (Days 1, 2, 4, 8, 16).	The longest longitudinal study (up to 480 days).
Liu 2023 (22)	330: Discovery (164) and Validation (166) set.	CoronaVac (Inactivated)	5 time points (before, during, and up to 30 days after the second dose).	Focus on metabolic switching (Warburg Effect).
Tang 2022 (23)	207 (Healthy adults aged 18–59). Median age 34 years.	BBIBP-CorV (Inactivated)	Day 0 (before the first dose), Day 14 (after the first dose), Day 42 (14 days after the second dose).	The study also examined correlations with the microbiome.
Tang 2024 (24)	70 (Young adults): CVS + Placebo (35) vs CVS + HSSD (35).	CoronaVac (CVS) (Inactivated) – Third dose (Booster).	Day 0 (Before the third dose), Day 14 (After the third dose).	The study concerned response modulation using traditional Chinese medicine (HSSD).
Wang 2022 (12)	50 (Healthy volunteers aged 21–56).	CoronaVac (Inactivated)	Before the first dose, approximately 21 days after the first dose, approximately 14 days after the second dose.	Analysis of both metabolomics and proteomics (Ig and Complement Cascade).
Zhang 2022 (25)	32 (Selected from 106): Selected based on extreme values (top/bottom 30%) of NAbs and BMI.	CoronaVac or BBIBP-CorV (Inactivated)	2–4 weeks (14–28 days) after the second dose.	Focus on predictive biomarkers of strong response (High-Antibody).

**Table 2:** Data synthesis.

Ref.	Effect on lipids	Impact on amino acids and energy metabolism	Key message
Abufares 2024 (19)	↑ Common pathways (in both types of vaccines): Sphingolipid metabolism	↑ Common pathways (in both types of vaccines): Histidine metabolism.	Neopterin (35× higher after Pfizer)- a biomarker of immune activation after mRNA. Syringic acid and 5-methoxytryptophol may indicate the activation of anti-inflammatory and antioxidant pathways after mRNA vaccination.
Dagla 2022 (11)	High Responders (HR): ↑ Higher Cholesterol and Phospholipids (HDL profile). Low Responders (LR): ↑ Higher Triacylglycerols and Ceramides (4 types).	HR (before the first dose): Higher levels of ↑: L-histidine, L-phenylalanine, L-glutamine, L-lysine. ↑ glutamine/valine ratio.	High amino acids prior to vaccination: Predictor of a strong response. Ceramides: Biomarker of low response and inflammation.
Ghini 2022 (20)	Naive: Significant reduction in LDL cholesterol/phospholipids and ApoB100. Greatest change in LDL5. Recovered patients: Minimal changes.	No significant changes in metabolites were observed in any group.	Metabolic changes (decrease in LDL) appeared after 7 days, intensified between 14 and 21 days, and partially disappeared after a month.
He 2022 (13)	↓ Phenylacetate, L-carnitine.	↑ L-glutamic acid, GABA, succinic acid, L-leucine.	Combination of GABA (↑) and Indole (↓): Distinction between vaccinated and unvaccinated individuals (AUC = 0.96). GABA correlates positively with IgG.
Lang 2025 (21)	Early response (Day 1–2): ↑ LPC(18:2), LPE(18:1), Cer(d18:1/24:1). ↓ PC(36:4), triacylglycerols. Late (Day 28): Return to normal, maintained ↑ sphingomyelin, LPC(18:2) in women.	Early response: ↑ Kynurenine, Glutamate, Histidine, Tryptophan. Pathways: Tryptophan–kynurenine and Glycerophospholipid/Sphingolipid Metabolism.	Kynurenine and LPC(18:2): Correlates with IgG. High Kynurenine/Tryptophan Ratio: Predictor of a strong humoral response.
Liu 2023 (22)	↑ levels of conjugated bile acids (e.g. TCA, TDCA) and unconjugated bile acids (GCA, CDCA).	↑ levels of amino acids (e.g. glutamic acid, phenylalanine). ↓ β-oxidation and the TCA cycle, increase in glycolysis.	Vaccination: Metabolic switching (Warburg effect) consistent with immune cell activation. ↑ Bile acids: Immune cell activation.
Tang 2022 (23)	No detailed data on changes in HDL/LDL/TG	↑ SCFA metabolism and related pathways (e.g. acetic acid, butyric acid, isovaleric acid).	SCFA (serum and faeces) positively correlate with antibody levels. *C. aerofaciens* and *V. dispar* strains (D0): Predictors of a strong response.
Tang 2024 (24)	↑ Lipid metabolism (e.g. methyl palmitate, SM, PC).	↑ Metabolism of purines, vitamin B6, folates, arginine and proline.	Modulation of these pathways by HSSD: Enhancement of humoral response (booster study with Chinese medicine).
Wang 2022 (12)	↑ Fatty acids. Altered biosynthesis of unsaturated fatty acids	↑ Most amino acids (e.g. valine, tryptophan, leucine). ↓ TCA cycle intermediates (pyruvate, citrate, malate, lactate).	TCA Cycle and Amino Acid Metabolism (Arginine, Proline, Phenylalanine, Tryptophan): Key pathways strongly linked to Humoral Response (IgG).
Zhang 2022 (25)	Metabolites of the Sphingolipid Signalling Pathway, e.g. SM(d18:0/18:1(11Z))	↑ Increase in Pregnenolone (x9.92) ↑ Pregnenolone metabolism (steroidogenesis)	↑ Elevated Pregnenolone and Sphingolipid Metabolites Associated with Higher Antibody Response (IgG and Total Ab).

**Table 3:** Summary of metabolomic findings.

Ref.	Metabolomic element	Effect
Dagla 2022, He 2022, Lang 2025, Liu 2023, Wang 2022 (11–13, 21, 22).	Amino acids (General)	Increased levels or strong correlation with the response for amino acids involved in providing building blocks for immune cells (e.g. glutamine/glutamate, phenylalanine, tryptophan).
Dagla 2022, Lang 2025, Liu 2023, Wang 2022 (11, 12, 21, 22).	Tryptophan/Phenylalanine pathway	The metabolism of tryptophan and phenylalanine is significantly modulated by vaccination, and its products are associated with the immune response.
Dagla 2022, He 2022, Lang 2025, Liu 2023 (11, 13, 21, 22).	Glutamine/Glutamate	Change in glutamine/glutamate levels after vaccination or correlation with a strong humoral response.
Abufares 2024, Dagla 2022, Lang 2025, Zhang 2022 (11, 19, 21, 25).	Sphingolipid Metabolism	The sphingolipid pathway is modulated after vaccination (increase or decrease in metabolites), and its changes are associated with the status of the immune response (HR/LR).
Liu 2023, Wang 2022 (12, 22).	Energy Metabolism (TCA)	A decrease in TCA cycle intermediates (e.g. citrate, pyruvate) in plasma is observed after vaccination, suggesting metabolic switching in activated immune cells (Warburg effect).
Dagla 2022, Lang 2025 (11, 21).	Response Prediction (HR/LR)	High baseline amino acid levels (e.g., histidine, glutamine, phenylalanine) prior to vaccination predicted a strong humoral response (High Responder).
Dagla 2022, Lang 2025 (11, 21).	Early Activation Markers	An early (1–2 days after dosing) increase in kynurenine levels and its ratio to tryptophan (kynurenine/tryptophan ratio) is a predictor of a strong humoral response.

Detailed metabolomic data at the study level and individual study conclusions are presented in **Table 2**. To account for the heterogeneity of results, **Table 3** presents a synthesis of results from different studies, highlighting consistent metabolic signatures that persist across different vaccine platforms and analytical methods.

### 3.2 Key metabolic findings

#### 3.2.1 Modulation of amino acid metabolism

Most studies have consistently shown that amino acid metabolism is one of the most dynamically modified pathways after vaccination (11–13, 21, 22).

Increase in overall amino acid levels: Elevated levels of numerous amino acids (valine, leucine, tryptophan, glutamine/glutamate) were found in plasma after vaccination (12, 22). This phenomenon reflects the increased demand of activated immune cells for building materials and energy substrates, consistent with their proliferation and activation.

Tryptophan/Phenylalanine pathway: The metabolism of tryptophan and phenylalanine and their derivatives (e.g., kynurenine) has been strongly linked to the immune response to vaccination (11, 12, 21, 22). In particular, an increase in kynurenine and the kynurenine/tryptophan ratio in the early phase after vaccination has been identified as a predictor of a strong humoral response (21).

#### 3.2.2 Shifts in energy and lipid metabolism

It has been demonstrated that vaccination induces characteristic shifts in cellular energy metabolism and modulates lipid pathways.

Warburg effect (energy switch): Studies on inactivated vaccines (CoronaVac) have shown a decrease in TCA cycle intermediates (e.g., citrate, pyruvate) with a simultaneous increase in glycolysis (12, 22). This phenomenon, analogous to the Warburg effect, is characteristic of activated lymphocytes and macrophages switching from aerobic respiration to anaerobic glycolysis.

Sphingolipid metabolism: Modulation of the sphingolipid pathway has been confirmed in studies on mRNA and inactivated vaccines (11, 19, 21, 25). Both sphingomyelins and ceramides have been shown to be involved in the immune response. Importantly, elevated ceramide levels prior to vaccination have been associated with a poor humoral response (Low Responders) (11).

#### 3.2.3 Early markers and response prediction

Baseline Amino Acid Levels: High plasma amino acid levels (e.g., histidine, glutamine, phenylalanine) measured on day 0 were correlated with achieving High Responder status after vaccination (11, 21).

Microbiome Markers: Studies on inactivated vaccines have shown that metabolites of microbiome origin, especially short-chain fatty acids (SCFA), positively correlate with antibody levels, indicating the role of the gut-immune axis (23).

### 3.3 Risk of bias

**Figure 2:**
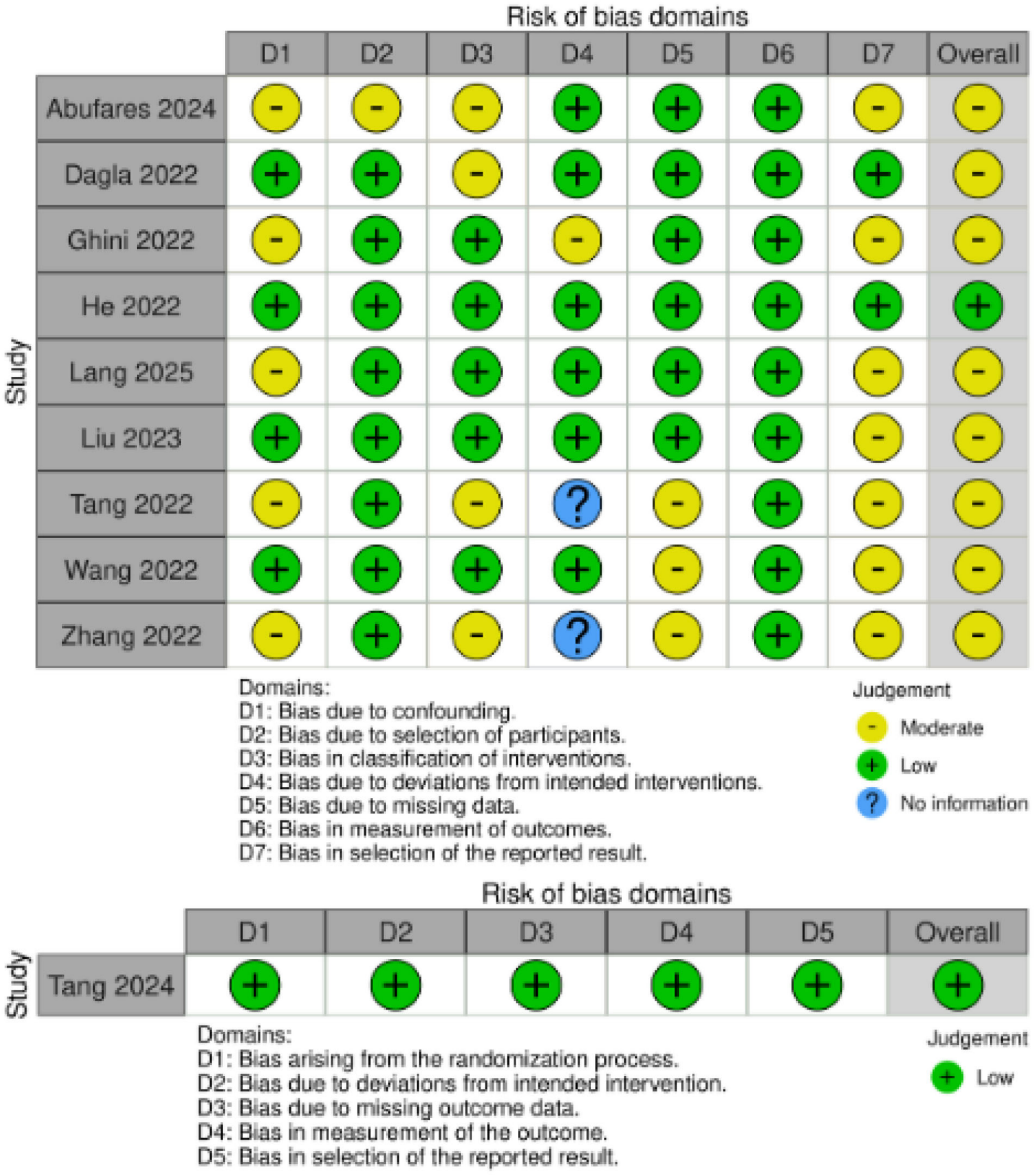
Risk of bias.

A key recurring inaccuracy in non-randomized studies was selection bias, where comparison groups were defined *post-hoc* based on the antibody levels achieved. This approach creates a fundamental bias in estimating the relationship between the metabolic biomarker and the immune response. A second significant weakness was insufficient control for confounding factors such as age, sex, or BMI. In studies where groups were unevenly distributed in terms of age and gender, the lack of statistical adjustment raised doubts about the reliability of the estimated differences in metabolism. In addition, metabolic analyses often contained inaccuracies related to missing data or non-standard data handling. Some key analyses were based on data with significant sample loss (even >70%) or used simplified imputation methods (e.g., 1/5 of the minimum value) for LC-MS data, which reduced the reliability of the results. There was also a risk of selective reporting of results, with researchers choosing the most striking metabolites (e.g., VLDL-TG, Pregnenolone) from hundreds of measured variables for discussion without relying on a pre-specified analysis plan. In contrast to the above, studies using a self-controlled (pre-post) design on homogeneous cohorts (12, 13, 22) demonstrated a stronger methodology in terms of controlling for baseline confounders. In contrast, the only randomized controlled trial analysed (24) did not show any significant inaccuracies, confirming its high methodological quality. The assessment of the risk of bias in specific publications is summarised in **Figure 2**.

## 4 Discussion

### 4.1 Key findings and interpretation

This systematic review, conducted in accordance with the PRISMA 2020 guidelines (14), synthesizes data from 10 studies involving more than 1,200 healthy participants, analysing metabolomic changes in venous blood after vaccination against COVID-19. The studies focused mainly on mRNA (Pfizer/Comirnaty) and inactivated (CoronaVac, BBIBP-CorV, Sinopharm) vaccines, excluding vector and subunit platforms due to a lack of eligible publications. Key findings indicate consistent, albeit heterogeneous, modifications in amino acid, lipid, and energy metabolism associated with immune system activation. These results complement earlier reports in vaccinology (9, 10), highlighting the role of the metabolome as an early indicator of immune response.

Despite differences in sensitivity between platforms (higher for LC-MS in the case of lipids, better repeatability for NMR in the case of major metabolites), the kynurenine pathway and changes in glutamine levels show high consistency in both techniques. These changes are most pronounced in the acute phase (1–2 days after vaccination), while lipoprotein profiles (detected mainly by NMR) evolve over a longer period (up to 21 days), suggesting that the early metabolic response is dominated by amino acid metabolism and the late response by lipid remodelling (26).

The complementary nature of NMR and LC-MS platforms is crucial for a comprehensive metabolomic landscape. While LC-MS offers higher sensitivity for low-abundance metabolites, 1H-NMR provides superior reproducibility and minimal sample preparation, as previously highlighted in the context of neuroimmunological disorders.

#### 4.1.1 Modulation of amino acid metabolism as a universal signature of immune activation

Most studies (11–13, 21, 22) have consistently shown an increase in amino acid levels (including glutamine/glutamate, phenylalanine, tryptophan) after vaccination, reflecting the increased demand of proliferating lymphocytes for energy and building substrates (10). In particular, the tryptophan-phenylalanine pathway, with an increase in kynurenine and the kynurenine/tryptophan ratio, has emerged as a predictor of a strong humoral response (11, 21). Mechanistically, kynurenine a product of tryptophan degradation via inducible indoleamine-2,3-dioxygenase (IDO) in activated macrophages and dendrites promotes immune tolerance but, in the context of vaccination, correlates with IgG production (27, 28). High baseline amino acid levels (histidine, glutamine) prior to vaccination predict “High Responder” status (11, 21), which is consistent with studies on influenza and yellow fever vaccines, where pre-vaccination amino acid profiles determine the strength of the response (10).

#### 4.1.2 Energy switching (Warburg effect) in response to inactivated vaccines

Studies with CoronaVac (12, 22) have documented a decrease in TCA cycle intermediates (citrate, pyruvate) with an increase in glycolysis, analogous to the Warburg effect in activated immune cells (29). This switch from aerobic respiration to anaerobic glycolysis supports rapid ATP production and biosynthesis in T and B lymphocytes (30). Similar changes were observed in metabolomics after SARS-CoV-2 infection (31), suggesting that vaccination mimics the early phases of the antigenic response without infectious pathology.

#### 4.1.3 The role of lipid metabolism in inflammatory regulation and response

The sphingolipid pathway was modulated in both vaccine platforms (11, 19, 21, 25), with an increase in sphingomyelins and ceramides. Ceramides prior to vaccination correlated with low responders and inflammation (11), confirming their proapoptotic and proinflammatory role in the immune system (32). In turn, lysophosphatidylcholine (LPC(18:2)) and kynurenine early (1–2 days) after mRNA correlated with IgG (21). Platform differences: mRNA induced neopterin (19) as a marker of IFN-γ activation, while inactivated mRNA affected bile acids and SCFA via the gut-immune axis (22, 23, 33).

#### 4.1.4 Response predictors and clinical implications

Baseline markers (amino acids, ceramides, SCFA, specific bacteria such as C. aerofaciens) enable stratification of the risk of poor response (10–20% of the population (5)). This paves the way for personalized interventions, e.g., microbiome modulation (23) or supplementation (24). In the long term (21), lipid changes persist in women, suggesting sexual dimorphism (34).

### 4.2 Comparison with broader literature

The results of this systematic review correspond to the broader context of metabolomic research on post-vaccination responses, both in relation to other vaccines and to the pathophysiology of SARS-CoV-2 infection. At the same time, they reveal specific features of COVID-19 vaccination resulting from unique technological platforms and the pandemic context.

#### 4.2.1 Consistency with classical models of vaccinology systems

Studies using the vaccinology systems approach (9, 10), have shown that early metabolomic changes after vaccination against influenza (trivalent inactivated) and yellow fever (live attenuated YF-17D) are highly correlative of subsequent humoral and cellular responses. In particular, the increase in kynurenine levels and the kynurenine/tryptophan ratio observed in the Lang 2025 study after the Comirnaty mRNA vaccine accurately reflects the analogous changes described after YF-17D (10). In both cases, activation of the IDO-1 pathway in antigen-presenting cells (APCs) leads to local depletion of tryptophan, which inhibits the proliferation of regulatory T cells (Tregs) and promotes Th1/Th17 differentiation—a key mechanism for generating immune memory (27, 28). Similarly, the increase in LPC(18:2) and sphingomyelin levels following mRNA (21) resembles the lipid profile following influenza vaccination, where lysophospholipids were markers of early dendritic cell activation (10). The activation of the kynurenine pathway observed post-vaccination mirrors broader metabolic shifts seen in other states of systemic inflammation and barrier dysfunction, where tryptophan-to-kynurenine conversion serves as a key regulatory node (35).

### 4.3 Limitations and future directions

Despite identifying consistent metabolic patterns, the study results are subject to limitations typical of metabolomics, such as selection bias (in the case of *post-hoc* definition of HR/LR groups) and insufficient control of confounding factors (age, gender, BMI).

A significant limitation of this review is the exclusion of vector-based (e.g., AstraZeneca) and protein subunit (e.g., Novavax) vaccines due to a lack of eligible metabolomics studies at the time of the search. Consequently, the identified signatures particularly the ‘Warburg-like’ switch seen in inactivated vaccines may not be fully generalizable to all technology platforms. While amino acid mobilization appears to be a conserved feature of general immune activation, it remains unclear whether the specific lipidomic shifts are platform-dependent or universal. Future research should fill this gap to determine a truly ‘universal’ metabolic code of vaccination.

Further prospective studies are needed combining metabolomics with proteomics and transcriptomics (which is already partially happening) to create a more complete picture of the molecular mechanisms of the immune response to vaccination. Prospective validation of biomarkers such as the Kyn/Trp ratio or baseline ceramide/amino acid levels for practical clinical use in identifying individuals at risk of poor response (Low Responders). Confirmation that metabolic changes induced by vaccination are transient and do not lead to permanent metabolic disorders.
